# Association between ethylene oxide exposure and osteoarthritis risk: an analysis of NHANES data (2013–2014 and 2017–2018)

**DOI:** 10.3389/fpubh.2025.1511215

**Published:** 2025-01-30

**Authors:** Zhongshan Li, Qi Qu, Zhiyu Wang, Shuanglin Mou, Rui Jiang, Wensheng Zhu

**Affiliations:** ^1^College of Acupuncture and Orthopedics, Hubei University of Chinese Medicine, Wuhan, China; ^2^Medical School, Hubei Minzu University, Enshi, China; ^3^Department of Osteology, Huanggang Hospital of Traditional Chinese Medicine Affiliated to Hubei University of Chinese Medicine, Huanggang, China

**Keywords:** osteoarthritis, ethylene oxide, epidemiology, NHANES, cross-sectional study

## Abstract

**Background:**

Ethylene oxide (EO) is widely used as a disinfectant and is also a common environmental pollutant. Exposure to EO has been associated with various systemic diseases, posing crucial health risks. However, EO is frequently employed as a sterilizing agent in orthopedics, while its association with the risk of skeletal system diseases remains insufficiently evaluated. This study aims to investigate the association between EO exposure and the risk of Osteoarthritis (OA), a prevalent orthopedic condition.

**Methods:**

A total of 3,386 participants were selected from the National Health and Nutrition Examination Survey (NHANES) 2013–2014 and 2017–2018 cycles, including 952 individuals with OA. Box plots assessed EO concentration differences between OA and non-OA groups. Weighted logistic regression models and restricted cubic spline (RCS) models were used to evaluate the relationship between EO exposure and OA risk. Subgroup analysis and interaction test explored variations in the association across different characteristics.

**Results:**

No significant difference in EO concentrations was found between OA and non-OA groups. In multivariate logistic regression, high EO level exposure was significantly associated with increased OA risk. Additionally, a nonlinear U-shaped and J-shaped association was observed in the unadjusted and adjusted RCS models, respectively. Subgroup analysis revealed that the association between EO exposure and OA risk was more pronounced in the 20–40 and 40–65 age groups, never smokers (Not at all), and those with low calcium levels (< 8.5 mg/dL) or low vitamin D levels (< 75 nmol/L).

**Conclusions:**

EO exposure is associated with OA risk, exhibiting a J-shaped relationship, with this association being particularly pronounced in individuals under 65 years old or those with low calcium or vitamin D levels. Further prospective studies are needed to examine the association between EO exposure and OA risk.

## 1 Introduction

Osteoarthritis (OA) is a prevalent orthopedic condition marked by the degeneration of articular cartilage and subchondral bone sclerosis (1). OA causes joint pain, deformities, and functional impairment, and is also associated with an increased risk of cardiovascular events, deep vein thrombosis of the lower extremities, and hip fractures, thereby significantly impacting patients' quality of life (2–5). Currently, there are over 300 million patients with OA worldwide, and the prevalence of OA is rising with the aging population (6). Previous studies have found risk factors for OA including aging, obesity, joint injury, gender, ethnicity, and socioeconomic status, among others (7, 8). Recent studies have revealed that environmental pollutants such as heavy metals, PM_2.5_, PM_10_, NO_2_, and SO_2_ can also increase the prevalence of OA (9–11). Therefore, it is crucial to identify and evaluate the impact of common environmental pollutant exposure on the risk of OA.

Ethylene oxide (EO), as a widely used industrial chemical for sterilizing various materials such as agricultural products, medical supplies, and hospital equipment, is also a prevalent distributed environmental pollutant (12). Humans are typically exposed to EO through working environments, air pollution, medications and hygiene products (13). EO has been identified to exhibit various acute and chronic toxicities, causing damage to the lungs, kidneys, central nervous system, and cardiovascular system in human, leading to multiple health issues (14–17). The International Agency for Research on Cancer of the World Health Organization has classified it as a Group 1 carcinogen (18). EO is commonly used for the sterilization of orthopedic instruments, materials, and medications, which are frequently used by individuals with orthopedic conditions (19, 20). However, it remains unclear whether EO exposure impacts orthopedic diseases. Therefore, this study utilized data from the National Health and Nutrition Examination Survey (NHANES) 2013–2014 and 2017–2018 cycles to explore the association between EO exposure and the prevalence of OA, a common orthopedic disease.

## 2 Methods

### 2.1 Study population

**Figure 1:**
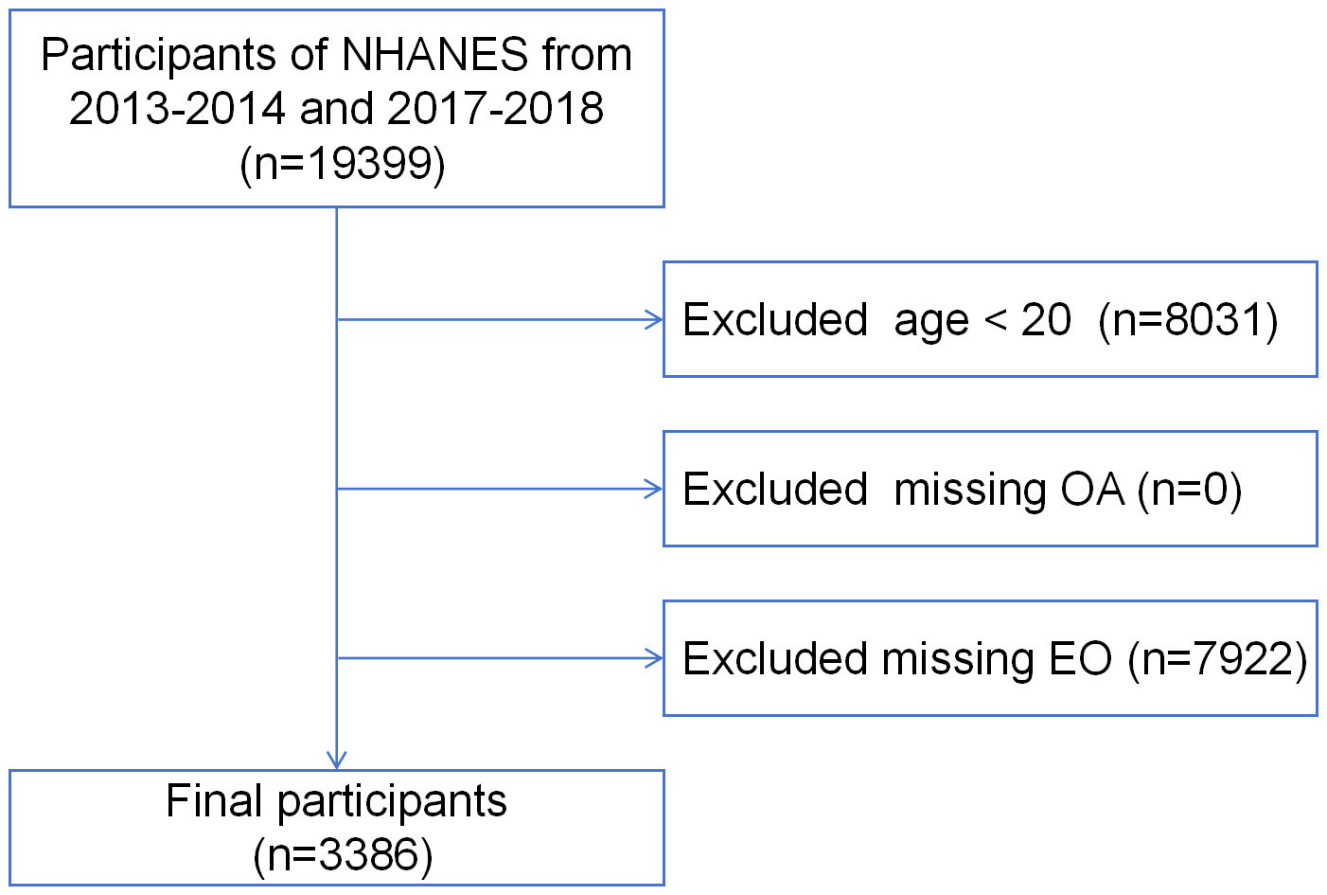
Flow chart of participants' enrollment process. NHANES, National Health and Nutrition Examination Survey; EO, ethylene oxide.

NHANES is a large-scale national survey led by the Centers for Disease Control and Prevention (CDC). It combines interviews, physical examinations, laboratory tests, and health behavior questionnaires to cover various health indicators and environmental exposure factors, assessing the health and nutritional status of the U.S. population. For this study, we selected 19,399 individuals from the 2013–2014 and 2017–2018 cycle. After excluding those younger than 20 years (*n* = 8,031), and those lacking OA questionnaire data (*n* = 0) or EO assay results (*n* = 7,922), a total of 3,386 participants were included in the final analysis (Figure 1). Since the research data was obtained from a public database (https://www.cdc.gov/nchs/nhanes/index.htm), ethical review was conducted by the CDC (https://www.cdc.gov/nchs/nhanes/irba98.htm).

### 2.2 Osteoarthritis

OA was determined by reviewing the medical conditions section (variable name prefix MCQ) in the self-reported personal interview data. Participants were asked two questions regarding OA: “Has a doctor or other health professional ever told you that you had arthritis?” and “Which type of arthritis was it?” A response of “Yes” to either question was recorded as a positive case of OA.

### 2.3 Measurement of ethylene oxide

According to the NHANES Laboratory Procedures Manual (LPM) (https://wwwn.cdc.gov/Nchs/Nhanes/2013-2014/ETHOX_H.htm), blood samples were collected and processed for testing at the National Center for Environmental Health. The testing procedure included the following steps: first, preparation of the specimen for measurement of hemoglobin adducts of ethylene oxide; second, measurement of total hemoglobin in the sample solution used for hemoglobin adduct measurements; third, modified Edman reaction in the sample solution and isolation of Edman products; lastly, analysis of Edman products by high-performance liquid chromatography coupled with tandem mass spectrometry (HPLC-MS/MS) and processing of results. The results were reported in pmol adduct per gram of hemoglobin. The entire process employed various quality controls to ensure the accuracy and precision of the test results.

### 2.4 Physical activity

MET-minute is an indicator used to measure the intensity and duration of physical activity. MET (Metabolic Equivalent of Task) represents the metabolic equivalent of an activity. MET-minute is calculated by multiplying the MET value of the activity by its duration (in minutes), and is used to quantify the total energy expenditure of an individual over a specific period. In this study, weekly MET-minute composite scores were used to assess physical activity levels, based on the NHANES suggested MET Scores, with the following calculation formula (21): (8.0 MET scores × minutes of vigorous work-related activity) + (4.0 MET scores × minutes of moderate work-related activity) + (4.0 MET scores × minutes of walking or bicycling for transportation) + (8.0 MET scores × minutes of vigorous leisure-time physical activity) + (4.0 MET scores × minutes of moderate leisure time [recreational] physical activity). According to the Physical Activity Guidelines for Americans, physical activity was divided into four categories (22): sedentary (no regular physical activity), insufficient (performing some regular activity, but < 500 MET-minutes per week), moderate (500–1,000 MET-minutes per week), and vigorous (more than 1,000 MET-minutes per week).

### 2.5 Other covariates

Demographic characteristics, including age, gender, ethnicity, education level, and family poverty income ratio (PIR), were extracted from the demographics file. PIR is defined as the ratio of family income to the poverty threshold, with participants categorized into low income (< 1.3), middle income (1.3–3.5), and high income (≥3.5) groups. Body mass index (BMI) was calculated as weight (kg) divided by height squared (m^2^), and classified into < 25 kg/m^2^ and ≥25 kg/m^2^ categories. Current smoker status was determined based on the response to the question, “Do you now smoke cigarettes?” Past-year alcohol drinking was categorized according to the response to “In the past 12 months, on those days that you drank alcoholic beverages, on average, how many drinks did you have?” into Non-drinker, 1–3 drinks, and ≥4 drinks groups. Diabetes mellitus and hypertension were determined based on medical diagnoses. Total calcium levels were classified according to clinical standards as insufficient (< 8.5 mg/dL) or sufficient (≥8.5 mg/dL). Similarly, 25-hydroxyvitamin D2+D3 levels were categorized as deficient (25–50 nmol/L), insufficient (50–75 nmol/L), and sufficient (≥75 nmol/L) (23).

### 2.6 Statistical analysis

NHANES employs a complex sampling design. This study used the subsample weight WTSA2YR for weighting calculations to correct for representativeness bias. To maintain data integrity, missing values in covariates were handled as follows: continuous variables with missing values below 10% were imputed with the mean if normally distributed and with the median if not, while continuous variables with missing values above 10% and all categorical variables were addressed using multiple imputation. All continuous variables were categorized, and results were presented as absolute values (n) or percentages (%), with chi-square tests used for evaluation. The skewed EO values were log10-transformed, analyzed using the Kruskal-Wallis test, presented as M (Q_1_, Q_3_), and then grouped into quartiles.

First, weighted univariate and multivariate logistic regression analyses were conducted to calculate odds ratios (ORs) and 95% confidence intervals (CIs) to explore the relationship between log10-transformed EO levels and OA risk. Potential confounders were adjusted to ensure the reliability of the results. The crude model did not adjust for any covariates. Model I adjusted for age, sex, race, PIR, and education level. Model II adjusted for all covariates. Next, restricted cubic splines (RCS) were used to explore potential complex nonlinear relationships between log10-transformed EO levels and OA risk, using the 10th, 50th, and 90th percentiles as knots. Finally, subgroup analysis and interaction test were then performed on potential confounding variables to examine the consistency of the relationship between EO and OA across different subgroups and to identify sources of variation. All statistical analyses were conducted using R software (version 4.4.1). *P*-value < 0.05 was considered statistically significant.

## 3 Results

### 3.1 Baseline characteristics of participants

**Figure 2:**
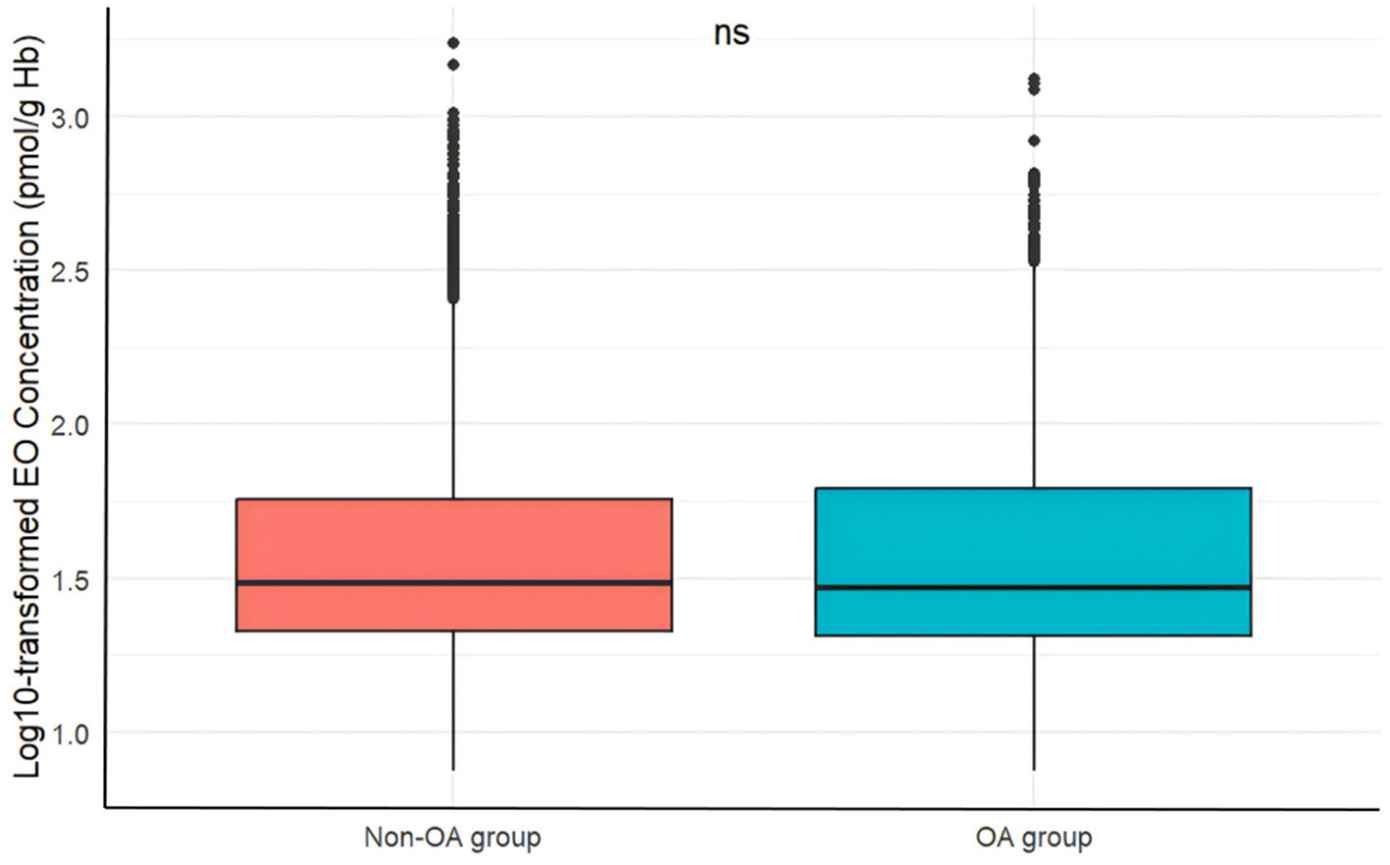
Comparison of log10-transformed EO concentration between OA and Non-OA Groups. EO, ethylene oxide; OA, osteoarthritis; ns: *P* > 0.05.

As detailed in Table 1, the study included 3,386 participants from the NHANES 2013–2014 and 2017–2018 cycles. Of these, 952 were diagnosed with OA, while 2,434 were not, yielding a weighted OA prevalence of 27.21%. Participants were categorized into four groups based on quartiles of log10-transformed EO levels: Q1 (0.87 ≤ log10 EO < 1.32 pmol/g Hb, *n* = 849), Q2 (1.32 ≤ log10 EO < 1.48 pmol/g Hb, *n* = 845), Q3 (1.48 ≤ log10 EO < 1.76 pmol/g Hb, *n* = 845), and Q4 (1.76 ≤ log10 EO ≤ 3.24 pmol/g Hb, *n* = 847). Significant differences were observed among groups regarding age, gender, ethnicity, education level, PIR, BMI, current smoker status, past-year alcohol drinking, diabetes mellitus, and 25-hydroxyvitamin D2+D3 levels (all *P* < 0.05). No meaningful differences were found for hypertension and total calcium levels. Additionally, Figure 2 shows no significant differences in EO concentrations between OA and non-OA participants (*P* > 0.05).

**Table 1:** Baseline characteristics of participants after imputing missing values according to quartiles of log10-transformed EO.

**Variables**	**Q1 (*n =* 849)**	**Q2 (*n =* 845)**	**Q3 (*n =* 845)**	**Q4 (*n =* 847)**	***P*-value**
Age, *n* (%), years					< 0.001
20–40	235 (27.68)	254 (30.06)	270 (31.95)	332 (39.20)	
40–65	346 (40.75)	373 (44.14)	358 (42.37)	394 (46.52)	
≥65	268 (31.57)	218 (25.80)	217 (25.68)	121 (14.29)	
Gender, *n* (%)					< 0.001
Female	469 (55.24)	476 (56.33)	423 (50.06)	361 (42.62)	
Male	380 (44.76)	369 (43.67)	422 (49.94)	486 (57.38)	
Ethnicity, *n* (%)					< 0.001
Mexican American	132 (15.55)	138 (16.33)	152 (17.99)	62 (7.32)	
Other Hispanic	87 (10.25)	96 (11.36)	72 (8.52)	42 (4.96)	
Non-Hispanic white	388 (45.70)	308 (36.45)	273 (32.31)	363 (42.86)	
Non-Hispanic black	140 (16.49)	156 (18.46)	147 (17.40)	261 (30.81)	
Non-Hispanic Asian	71 (8.36)	121 (14.32)	173 (20.47)	64 (7.56)	
Other race	31 (3.65)	26 (3.08)	28 (3.31)	55 (6.49)	
Education level, *n* (%)					< 0.001
< 12th grade	147 (17.31)	155 (18.34)	179 (21.18)	225 (26.56)	
High school	200 (23.56)	179 (21.18)	171 (20.24)	260 (30.70)	
College or more	502 (59.13)	511 (60.47)	495 (58.58)	362 (42.74)	
PIR, *n* (%)					< 0.001
< 1.3	206 (24.26)	235 (27.81)	245 (28.99)	416 (49.11)	
1.3–3.5	348 (40.99)	297 (35.15)	311 (36.80)	289 (34.12)	
≥3.5	295 (34.75)	313 (37.04)	289 (34.20)	142 (16.77)	
BMI (kg/m^2^)					< 0.001
< 25.0	180 (21.20)	210 (24.85)	257 (30.41)	290 (34.24)	
≥25.0	669 (78.80)	635 (75.15)	588 (69.59)	557 (65.76)	
Physical activity, *n* (%), MET-min					< 0.001
< 500	504 (59.36)	527 (62.37)	553 (65.44)	465 (54.90)	
500–1,000	132 (15.55)	140 (16.57)	117 (13.85)	91 (10.74)	
≥1,000	213 (25.09)	178 (21.07)	175 (20.71)	291 (34.36)	
Current smoker status, *n* (%)					< 0.001
Not at all	626 (73.73)	604 (71.48)	582 (68.88)	186 (21.96)	
Some days	59 (6.95)	52 (6.15)	84 (9.94)	89 (10.51)	
Every day	164 (19.32)	189 (22.37)	179 (21.18)	572 (67.53)	
Past-year alcohol drinking, *n* (%)					< 0.001
Non-drinker	338 (39.81)	349 (41.30)	356 (42.13)	192 (22.67)	
1–3 drinks	365 (42.99)	352 (41.66)	322 (38.11)	395 (46.64)	
≥4 drinks	146 (17.20)	144 (17.04)	167 (19.76)	260 (30.70)	
Diabetes mellitus, *n* (%)					< 0.001
Yes	97 (11.43)	119 (14.08)	148 (17.51)	96 (11.33)	
No	723 (85.16)	700 (82.84)	656 (77.63)	731 (86.30)	
Borderline	29 (3.42)	26 (3.08)	41 (4.85)	20 (2.36)	
Hypertension, *n* (%)					0.465
Yes	339 (39.93)	310 (36.69)	312 (36.92)	328 (38.72)	
No	510 (60.07)	535 (63.31)	533 (63.08)	519 (61.28)	
Total calcium, *n* (%), mg/dL					0.094
< 8.5	6 (0.71)	2 (0.24)	4 (0.47)	10 (1.18)	
≥8.5	843 (99.29)	843 (99.76)	841 (99.53)	837 (98.82)	
25(OH)D, *n* (%), nmol/L					< 0.001
< 50	242 (28.50)	205 (24.26)	250 (29.59)	312 (36.84)	
50–75	293 (34.51)	290 (34.32)	315 (37.28)	321 (37.90)	
≥75	314 (36.98)	350 (41.42)	280 (33.14)	214 (25.27)	
Log10 EO, M (Q1, Q3), pmol/g Hb	1.23 (1.17, 1.28)	1.40 (1.36, 1.44)	1.57 (1.52, 1.64)	2.28 (2.05, 2.48)	< 0.001

EO, ethylene oxide; PIR, family poverty income ratio; BMI, body mass index; MET-min, MET-minutes; 25(OH)D, 25-hydroxyvitamin D2+D3; Log10 EO, log10-transformed EO; M, Median, Q1, 1st Quartile, Q3, 3st Quartile.

### 3.2 Weighted logistic regression analysis between EO and OA

Weighted univariate logistic regression analysis indicated that the association between log10-transformed EO and OA was not statistically significant in Supplementary Table 1, (OR = 1.18, 95%CI: 0.91, 1.52, *P* = 0.2). However, weighted multivariate logistic regression analysis revealed a significant association between EO and OA after adjusting for covariates. As shown in Table 2, In both Model 1 and Model 2, log-10 transformed EO was positively associated with the risk of OA (Model 1: OR = 1.54, 95%CI: 1.16–2.05, *P* = 0.005; Model 2: OR = 1.76, 95%CI: 1.12–2.77, *P* = 0.025). Furthermore, the risk was significantly higher in the Q4 group compared to the Q1 group (Q4 in Model 1: OR = 1.74, *P* = 0.003; Q4 in Model 2: OR = 2.02, *P* = 0.052), suggesting that higher EO concentration exposure may increase the risk of OA. In addition, trend analyses indicated a significant association between log-10 transformed EO concentration and OA risk in both Model 1 (*P* for trend = 0.014) and Model 2 (*P* for trend = 0.041).

**Table 2:** Weighted multivariate logistic regression analysis of log10-transformed EO for risk of OA.

	**Crude Model**		**Model I**		**Model II**	
	**Crude OR (95%CI)**	***P*-value**	**Adjusted OR (95%CI)**	***P*-value**	**Adjusted OR (95%CI)**	***P*-value**
Log10 EO	1.18 (0.91, 1.52)	0.2	1.54 (1.16, 2.05)	0.005	1.76 (1.12, 2.77)	0.025
Q1	Reference		Reference		Reference	
Q2	0.89 (0.69, 1.13)	0.3	0.93 (0.65, 1.32)	0.7	0.87 (0.43, 1.76)	0.5
Q3	0.95 (0.64, 1.42)	0.8	1.08(0.71, 1.64)	0.7	1.08 (0.47, 2.52)	0.7
Q4	1.14 (0.83, 1.57)	0.4	1.74 (1.24, 2.45)	0.003	2.02 (0.98, 4.16)	0.052
*P* for trend	0.5		0.014		0.041	

The crude model was not adjusted for covariates. Model I was adjusted for age, gender, ethnicity, PIR and Education level. Model II was adjusted for all covariates. OR, odd ratio; CI, confidence interval; EO, ethylene oxide; Log10 EO, log10-transformed EO.

### 3.3 Non-linear association between EO and the risk of OA

**Figure 3:**
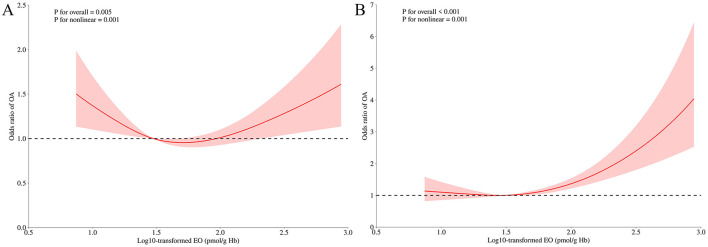
Odds ratio of OA according to log10-transformed EO levels in the overall population. The solid line and shadow represented the odds ratio of OA and 95% confidence interval, respectively. **(A)** no covariates were adjusted. **(B)** all covariates were adjusted. EO, ethylene oxide; OA, osteoarthritis.

The RCS model further investigated the non-linear relationship between EO and the risk of OA. Figure 3A reveals a U-shaped nonlinear association between log-10 transformed EO and OA risk in the unadjusted model (*P* for overall < 0.001, *P* for non-linearity = 0.001). Figure 3B demonstrates a J-shaped non-linear relationship between log-10 transformed EO and OA risk after adjusting for all confounding variables (*P* for overall < 0.001, *P* for non-linearity = 0.001).

### 3.4 Subgroup analysis

**Figure 4:**
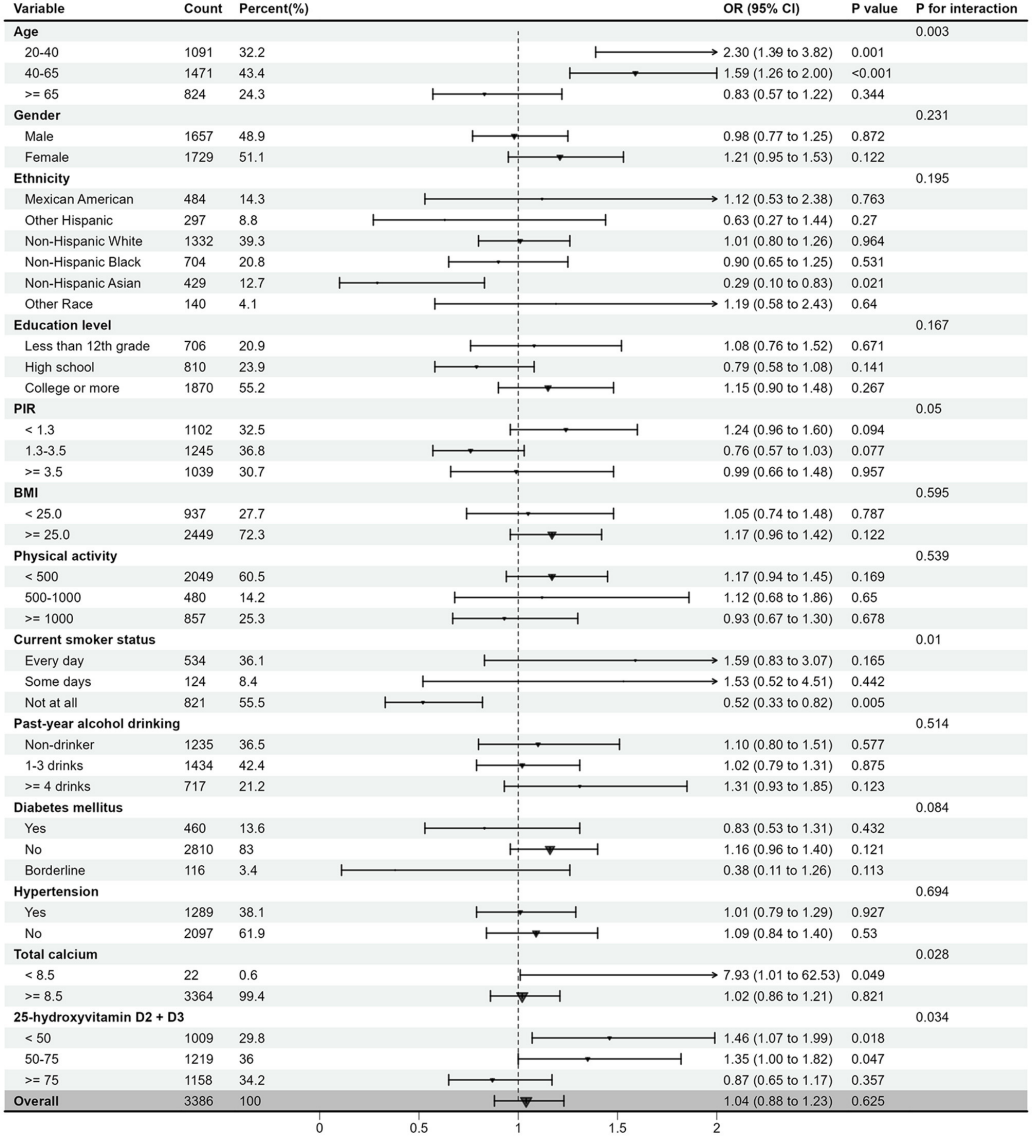
The relationship between log10-transformed EO and risk of OA according to different subgroups. EO, ethylene oxide; OA, osteoarthritis; OR, odds ratio; CI, confidence interval; PIR, family poverty income ratio; BMI, body mass index; MET-min, MET-minutes; 25(OH)D, 25-hydroxyvitamin D2+D3.

Figure 4 presents the results of the stratified analysis and interaction test for all covariates. Overall, the association between EO exposure and OA risk was not significant. However, in specific subgroups, EO exposure was strongly associated with either a positive or negative risk of OA. For instance, in the 20–40 and 40–65 age groups, as well as in groups with low calcium and low 25-hydroxyvitamin D2 + D3 levels, EO exposure was associated with a increased OA risk. Conversely, in never smokers (Not at all), EO exposure was linked to a decreased OA risk. Interaction tests indicated that age, smoking status, total calcium level, and 25-hydroxyvitamin D2 + D3 level are potential modifiers of the relationship between EO exposure and OA risk (all *P* < 0.05), suggesting these factors may influence the impact of EO exposure on OA risk.

## 4 Discussion

OA is a common and poorly treatable orthopedic condition affecting approximately 300 million people worldwide (6). Given the irreversible nature of cartilage damage, early prevention is crucial (24). EO is known for its multi-organ toxicity and presents a high exposure risk in the orthopedic field (13, 19, 20). This study utilized data from NHANES involving 3,386 participants to investigate the association between EO exposure and OA risk, and to assess the heterogeneity of this association with demographic and lifestyle variables. We found that while there was no significant difference in EO concentrations between OA and non-OA groups, high EO levels, particularly in the fourth quartile, were significantly associated with OA risk after adjusting for confounding factors. Additionally, a non-linear J-shaped association was observed. Moreover, this association was influenced by age, current smoker status, total calcium and 25-hydroxyvitamin D2 + D3 levels.

In our findings, the univariate RCS model revealed a U-shaped relationship between EO exposure and OA risk, suggesting that OA risk was higher at both low and high levels of EO exposure, while moderate exposure was associated with lower risk. After adjusting for covariates in the multivariate model, the curve shifted from a U-shape to a J-shape. This indicates that certain confounding factors may have masked the true relationship between EO exposure and OA risk in the univariate model. In the adjusted model, the negative impact of high EO exposure became more pronounced, while the effect of low exposure could potentially be attributed to other factors. Insights from univariate logistic regression and subgroup analyses suggest that these effects might be influenced by variables such as age, BMI, hypertension, and total calcium levels. Overall, when considering the results of multivariate logistic regression and the multivariate RCS model, it can be concluded that high levels of EO exposure have a significant impact on OA risk.

OA is associated with various environmental pollutants (9–11). However, no studies have yet evaluated the relationship between EO exposure and OA risk. Our study found a significant association between increased EO concentrations and elevated OA risk. Although the mechanisms underlying OA are not fully understood, factors such as age, obesity, inflammation, immunity, and genetics are known to be related to its development (25, 26). EO is known to exacerbate cardiovascular and respiratory diseases by increasing inflammation and oxidative stress (14, 15, 27). Additionally, EO can also react directly with macromolecules (DNA, RNA, or proteins), leading to somatic mutations and genotoxicity (28). Thus, we hypothesize that EO exposure may influence OA risk through the aforementioned mechanisms, but further research is needed to confirm this.

Regarding the observed association restricted to high levels of EO exposure, we propose several hypotheses. Low levels of EO exposure might trigger adaptive mechanisms or immune responses, providing temporary protective effects. However, when exposure exceeds a critical threshold, these mechanisms may be disrupted, leading to a sharp increase in OA risk. High EO exposure could also result in cumulative toxic effects, such as increased oxidative stress, inflammation, or cellular damage, substantially elevating OA risk. Additionally, the univariate model may not have fully accounted for the effects of key covariates, such as age distribution or comorbidities, which could exaggerate the OA risk associated with low exposure levels. These hypotheses warrant further investigation.

The subgroup analysis suggests that EO exposure has a positive impact on individuals under 65, while the effect is not significant in those aged 65 and older. This suggests that EO exposure may be a potential risk factor for osteoarthritis in younger populations, likely related to their more frequent social activities, which increase exposure opportunities (29). Future studies should explore the biological mechanisms underlying the differences in EO exposure across age groups. Additionally, in individuals with lower calcium and 25-hydroxyvitamin D2 + D3 levels, EO exposure may increase the risk of OA, suggesting that the impact of EO on OA is more pronounced in a low-calcium or low-vitamin D environment. One study confirmed that serum calcium has a negative causal effect on OA (30). Additionally, Saengsiwaritt et al. proposed that vitamin D may restore chondrocyte function and viability in knee osteoarthritis (KOA) by regulating autophagy in human chondrocytes (31). These findings indicated that low levels of calcium and vitamin D are associated with OA, and increasing the intake of calcium and vitamin D may help mitigate the negative impact of EO concentrations on osteoarthritis. Furthermore, it is noteworthy that existing studies have identified smoking as a risk factor for OA (32). However, our research suggests no association between EO exposure and OA risk among smokers. Conversely, in never smokers, individuals with high EO levels appeared to have a low risk of OA. Additionally, univariate logistic regression analysis revealed that non-smokers had a 25% increased risk of OA compared to smokers, contradicting current findings. This unreasonable result may be due to errors caused by missing data. Therefore, adequate levels of calcium and vitamin D may play a protective role or mitigate the effects of EO exposure in the prevention of osteoarthritis.

Our study has three strengths. First, this study is the first to reveal the association between EO exposure and the risk of OA. Second, a large sample size from the NHANES database was utilized. Third, explore the effects of EO exposure on different subgroups, providing deeper insights into the mechanisms underlying OA development. Nonetheless, some significant limitations are unavoidable. First, due to its cross-sectional design, it cannot establish a causal relationship between EO exposure and OA risk. Second, OA is a chronic progressive disease influenced by various environmental factors and genetic backgrounds. However, EO exposure data were obtained from a single measurement, which may not comprehensively reflect individuals' long-term exposure levels. Therefore, future research should employ longitudinal designs to validate the long-term health effects of EO exposure further. Finally, although this study controlled for multiple confounding factors, unmeasured confounders, such as dietary patterns and occupational exposures, may still influence the analysis results. Thus, further investigation including these factors is needed.

## 5 Conclusions

In conclusion, high levels of EO exposure are significantly associated with an increased risk of OA, demonstrating a J-shaped relationship. EO exposure may significantly elevate OA risk in individuals younger than 65, or those with low calcium or vitamin D levels. This finding provides empirical support for controlling EO exposure and improving bone health. Further prospective studies are needed to confirm our findings.

## Data Availability

Publicly available datasets were analyzed in this study. This data can be found here: https://www.cdc.gov/nchs/nhanes/?CDC_AAref_Val=https://www.cdc.gov/nchs/nhanes/index.htm.
