# Supplementary material for: Association between ethylene oxide exposure and osteoarthritis risk: an analysis of NHANES data (2013–2014 and 2017–2018)
Source: Front Public Health. 2025 Jan 30;13:1511215. doi: 10.3389/fpubh.2025.1511215 (PMC11822445; doi:10.3389/fpubh.2025.1511215)
Supplement: Supplementary file 1 [file Supplementary_file_1.pdf]

Supplementary file

Supplementary Table 1 Weighted univariate logistic regression analysis of risk factors associated with OA

**Variables**	**OR**	**95% CI**	***P*-value**
Log10 EO, pmol/g Hb	1.18	0.91, 1.52	0.2
Age, n (%), years			
20-40		Reference	
40-65	7.63	5.04, 11.6	<0.001
>= 65	20.6	13.9, 30.5	<0.001
Gender, n (%)			
Female		Reference	
Male	1.52	1.24, 1.87	<0.001
Ethnicity, n (%)			
Mexican American		Reference	
Other Hispanic	1.09	0.79, 1.51	0.6
Non-Hispanic White	2.74	1.99, 3.78	<0.001
Non-Hispanic Black	2.06	1.37, 3.12	0.001
Non-Hispanic Asian	0.87	0.61, 1.24	0.4
Other Race	3.64	1.94, 6.84	<0.001
Education level, n (%)			
Less than 12th grade		Reference	
High school	1.12	0.81, 1.55	0.5
College or more	1.04	0.79, 1.36	0.8
PIR, n (%)			
< 1.3		Reference	
1.3-3.5	0.90	0.69, 1.17	0.4
>= 3.5	1.04	0.81, 1.34	0.8
BMI (kg/m^2)			
< 25.0		Reference	
>= 25.0	2.23	1.69, 2.95	<0.001
Physical activity, n(%), MET-min			
< 500		Reference	

Variables	OR	95% CI	*P*-value
500-1000	0.69	0.51, 0.94	0.020
>= 1000	0.68	0.51, 0.91	0.012
Current smoker status, n(%)			
Every day		Reference	
Some days	0.69	0.39, 1.23	0.2
Not at all	1.25	1.01, 1.56	0.040
Past-year alcohol drinking, n(%)			
Non-drinker		Reference	
1-3 drinks	0.66	0.51, 0.84	0.002
>= 4 drinks	0.50	0.35, 0.71	<0.001
Diabetes mellitus, n(%)			
Yes		Reference	
No	0.35	0.26, 0.47	<0.001
Borderline	0.96	0.55, 1.69	0.9
Hypertension, n(%)			
Yes		Reference	
No	0.27	0.22, 0.32	<0.001
Total calcium, n(%), mg/dL			
< 8.5		Reference	
>= 8.5	1.03	0.31, 3.41	>0.9
25(OH)D, n(%), nmol/L			
< 50		Reference	
50-75	1.31	0.96, 1.79	0.085
>= 75	3.38	2.39, 4.77	<0.001

OR, odd ratio; CI, confidence interval; EO, ethylene oxide; PIR, family poverty income ratio; BMI, body mass index; MET-min, MET-minutes; 25(OH)D, 25-hydroxyvitamin D2+D3.
